# Patient and health worker perspectives on quality of HIV care and treatment services in Haiti

**DOI:** 10.1186/s12913-023-09041-2

**Published:** 2023-01-23

**Authors:** Nancy Puttkammer, Joseph Adrien Emmanuel Demes, Witson Dervis, Jean Marcxime Chéry, Josette Elusdort, Elizabeth Haight, Jean Guy Honoré, Jane M. Simoni

**Affiliations:** 1 International Training and Education Center for Health (I-TECH), Department of Global Health, University of Washington, 325 Ninth Ave, Box # 359932, Seattle, WA 98104 USA; 2 Faculté de Médecine et de Pharmacie, Université d’Etat d’Haïti (National University of Haiti), 89, Rue Oswald DURAND, Port-Au-Prince, HT6110 Haïti; 3 Centre Haïtien de Renforcement du Système Sanitaire (CHARESS), 14, Route de Jacquet, Delmas 95, Port-Au-Prince, Haïti; 4 Department of Psychology, University of Washington, 3921 W Stevens Way NE, Box #351525, Seattle, WA 98195-0000 USA

**Keywords:** HIV/AIDS, Haiti, Antiretroviral therapy, Adherence, Retention in care, Cultural factors in healthcare

## Abstract

**Background:**

Poor quality of care is a barrier to engagement in HIV care and treatment in low- and middle-income country settings. This study involved focus group discussions (FGD) with patients and health workers in two large urban hospitals to describe quality of patient education and psychosocial support services within Haiti’s national HIV antiretroviral therapy (ART) program. The purpose of this qualitative study was to illuminate key gaps and salient “ingredients” for improving quality of care.

**Methods:**

The study included 8 FGDs with a total of 26 male patients and 32 female patients and 15 smaller FGDs with 57 health workers. The analysis used a directed content analysis method, with the goal of extending existing conceptual frameworks on quality of care through rich description.

**Results:**

Dimension of safety, patient-centeredness, accessibility, and equity were most salient. Patients noted risks to privacy with both clinic and community-based services as well as concerns with ART side effects, while health workers described risks to their own safety in providing community-based services. While patients cited examples of positive interactions with health workers that centered their needs and perspectives, they also noted concerns that inhibited trust and satisfaction with services. Health workers described difficult working conditions that challenged their ability to provide patient-centered services. Patients sought favored relationships with health workers to help them navigate the health care system, but this undermined the sense of fairness. Both patients and health workers described frustration with lack of resources to assist patients in dire poverty, and health workers described great pressure to help patients from their “own pockets.”

**Conclusions:**

These concerns reflected the embeddedness of patient – provider interactions within a health system marked by scarcity, power dynamics between patients and health workers, and social stigma related to HIV. Reinforcing a respectful and welcoming atmosphere, timely service, privacy protection, and building patient perception of fairness in access to support could help to build patient satisfaction and care engagement in Haiti. Improving working conditions for health workers is also critical to achieving quality.

## Background

Quality of care is a key driver of health care utilization and health outcomes. In low- and middle-income countries (LMICs), a larger share of mortality is attributable to poor quality of services than to non-utilization of healthcare services, among causes of death amenable to healthcare intervention [26, 32]. HIV/AIDS is an area of both health burden and high investment to strengthen health systems in LMICs. In 2020, there were 37.7 million people living with HIV (PLHIV) globally, with 73% enrolled on life-saving antiretroviral treatment (ART) [44]. In the last two decades, approximately 20% of all development assistance focusing on health systems strengthening has targeted HIV/AIDS services, equaling $1.4 billion USD in 2020 [23]. Poor quality of care is a barrier to engagement in HIV care and treatment in LMIC settings [4, 6], so successfully controlling the global HIV pandemic will require further improvements in HIV quality of care.

Haiti has an adult HIV prevalence of approximately 2.0% [21] and the largest number of PLHIV in the Caribbean region [45]. Access to ART has rapidly expanded since the early 2000s [25, 40] such that by the end of 2020 80% of PLHIV were on ART and 68% had achieved HIV viral suppression [33, 45]. In a survey of coping and ART adherence among adult ART patients in Port-au-Prince, Rubens et al. [39] identified a positive association between satisfaction with care and ART adherence [39]. Haiti’s Ministry of Public Health and Population (MSPP) has documented patient concerns about privacy protection and stigma in healthcare, especially for adolescents, which could inhibit satisfaction with and engagement in HIV care and treatment [29].

Our understanding of quality of care is based upon several seminal models. Donabedian’s framework defines structure, process, and health outcomes as key dimensions for measuring healthcare quality [9, 10]. The National Academy of Sciences further specifies dimensions of quality, focusing on safety, effectiveness, patient-centeredness, accessibility (including timeliness and affordability), efficiency, and equity (see Table 1) [32]. Hanefeld’s work on healthcare quality and service utilization underscores that patient perceptions of quality drive service utilization, that perceived responsiveness of health services and perceived trust in the health system are important aspects of patient-centered services, that patient experience of quality develops over time, and that patient experiences at the individual level in interacting with health workers (HWs) are embedded within broader health system structures and community norms [18].

**Table 1:** Definitions of quality of care dimensions [32]

**Dimension**	**Definition**
**Safety**	“Avoiding harm to patients from the care that is intended to help them.”
**Effectiveness**	“Providing services based on scientific knowledge to all who could benefit, and refraining from providing services to those not likely to benefit (that is, avoiding both overuse of inappropriate care and underuse of effective care).”
**Patient-centeredness**	“Providing care that is respectful of and responsive to individual preferences, needs, and values and ensuring that people’s values guide all clinical decisions. Care transitions and coordination should not be centered on health care providers, but on recipients.”
**Accessibility, timeliness, affordability**	“Reducing unwanted waits and harmful delays for both those who receive and those who give care; reducing access barriers and financial risk for patients, families, and communities; and promoting care that is affordable for the system.”
**Efficiency**	“Avoiding waste, including waste of equipment, supplies, ideas, and energy, and including waste resulting from poor management, fraud, corruption, and abusive practices. Existing resources should be leveraged to the greatest degree possible to finance services.”
**Equity**	“Providing care that does not vary in quality because of personal characteristics such as gender, ethnicity, race, geographic location, and socioeconomic status.”

Understanding key gaps and levers for improving quality in an applied way, such as to improve HIV outpatient care in the Haitian context, is complex. In a systematic review of structure and process factors affecting quality of HIV outpatient care, Engelhard concludes that the studies were too diverse in methods, definitions and findings to explain drivers of quality or guide policies regarding standards of care [12]. Prior work in Haiti has demonstrated widely variable facility-level performance in compliance with HIV care guidelines (e.g. timely uptake of HIV viral load monitoring) and intermediate outcomes (e.g. ART retention after 6 months) [2, 31]. However, standard health service readiness indicators have had limited explanatory power in terms of illuminating key ingredients for achieving favorable health outcomes such as retention on ART [27].

Given the lack of clear explanatory evidence on salient “ingredients” for improving quality, qualitative research can illuminate actionable themes. This study reports on formative qualitative research on patient and HW perceptions of quality in ART service delivery, with a focus on the dimensions of safety, patient-centeredness, accessibility, and equity. The purpose of the research is to describe the specific nature of gaps in HIV care quality in Haiti, in order to guide pragmatic approaches to improving quality as a strategy to improve patient satisfaction and care engagement.

## Methods

### Study design

The present study was part of the formative phase of a research project to develop and test the *InfoPlus Adherence* intervention. This intervention worked at the level of the healthcare organization, combining two components: 1) an electronic medical record (EMR)–based alert to signal patients at high, medium or low risk of future ART treatment failure (based on a prediction model using existing socio-demographic, clinical, laboratory and ART adherence data); and 2) brief person-centered ART adherence counseling from the multidisciplinary healthcare team, informed by the EMR-based alert [36, 37].

During the formative phase of the project, we carried out qualitative focus group discussions (FGD) with ART patients and HWs, and the present study reports on their perspectives about quality of care within Haiti’s ART program. We used the Situated Information-Motivation-Behavioral Skills (IMB) Model of Care Initiation and Maintenance (sIMB-CIM), a well-described and validated model for ART adherence, to inform the question guide for the FGD [14, 41]. The question guide focused on how the ART program in Haiti fosters information, motivation and behavioral skills through patient education and psychosocial support services. Our qualitative study identified themes related to quality of ART patient education and psychosocial support services, and we grouped these themes post hoc by applying the dimensions of quality in the National Academy of Sciences framework in our interpretation of the data.

### Study setting

With a per-capita GDP of $1,272 USD, Haiti is the poorest country in the Americas [47]. The life expectancy in Haiti is 63 years of age [46] and poverty-related causes of death and disability, including communicable diseases, maternal and neonatal disorders, and nutrition-related disorders, remain pervasive [30].

Haiti’s health system includes 1,007 health facilities, including referral hospitals, regular hospitals, health centers with beds, health centers without beds, and dispensaries. Much of the evidence on structure and process indicators of care quality come from the Service Provision Assessment (SPA) survey, carried out in Haitian health facilities using a census sampling approach in 2013 and 2017–18. Based on the 2013 SPA data, Gage et al. [17] developed a composite score for primary care quality encompassing domains of accessibility, effectiveness, management and organization, and primary care functions, finding that while 91% of the population lived within 5 km of a health facility, only 23% of the population lived within 5 km of good quality primary care [17]. In the 2017–18 SPA survey, only 42% of health facilities offered all elements considered to be part of the national package of primary care services (STI, acute care for childhood illness, prenatal care, family planning, childhood immunizations, and child growth monitoring), only 86% had access to an improved water source, only 76% had regular electricity, and only 52% had access to a computer with internet connectivity [22].

The present qualitative study was conducted within two large, public-sector, tertiary teaching hospitals, one in Port-au-Prince and the other in Cap Haïtien, the two largest urban areas in Haiti. Both sites are located in dense urban centers and serve a patient population of poor and working class urban residents as well as some patients from outlying semi-urban or rural areas. These sites typically enrolled 15–20 new ART patients per month. At the hospital in Port-au-Prince, the staff included 7–8 psychologists and social workers (for approximately 7,500 ART patients), while at the hospital in Cap Haïtien, the staff included no psychologists and two social workers (for approximately 6,500 ART patients). Both programs hosted community-based HIV services, supervised by nurses and including lay community health workers (CHWs), as part of differentiated HIV services [11]. These services include community-based ART distribution (known by its French-language acronym, DAC) and multi-month dispensing (MMD). With DAC services, on a voluntary basis, clinically stable ART patients can elect that CHWs deliver ART refills to the patient’s home or to a discreet location in the community. MMD refers to dispensing of 2–6 months’ worth of ART medications to stable ART patients, rather than requiring ART refills on a monthly basis.

### Participant recruitment and study procedures

The study team organized 4 focus group discussions (FGD) with male patients, 4 FGD with female patients and 15 smaller FGD with healthcare workers, based on a purposive sampling approach, as described in an earlier publication [35]. To recruit patients for FGD, clinic staff reviewed their patient registry to identify clients who met eligibility criteria (aged 18 and over, having started ART within the past 5 years, and having experienced some challenges with ART adherence according to provider report) and purposive sampling criteria of age and gender. They then contacted eligible clients either during their routine visits to the clinic or by telephone to invite them to participate and ceased outreach after reaching the desired number of participants for each FGD. Each group had 6–10 participants. Patients were grouped by sex and age (under vs. over 30 years), to ensure comfort and avoid power differentials. The sessions with HWs included 2–9 participants per group, with grouping by cadre (including registration and data clerks, triage nurses and clinical nurses, doctors, CHWs, laboratory personnel, pharmacy personnel, and psychologists/social workers). Using a semi-structured discussion guide, the FGD facilitator asked participants to discuss patient education, ART adherence counseling, support services, and patient-provider communication in the context of outpatient HIV services. FGDs were facilitated by a Haitian qualitative researcher in Haitian Kreyòl and French and took place in private meeting rooms within the hospitals. All interviews were audio recorded, transcribed, and translated into French by a professional, and reviewed for accuracy by study team members who observed the FGDs. Patients received a payment of $10 and HWs received a payment of $15 as compensation for their time and transportation costs to attend the interviews.

### Analysis

The analysis used a directed content analysis method, with the goal of extending existing conceptual frameworks on quality of care through rich description [20]. This method uses a naturalistic approach that combines deductive and inductive aspects in codebook development and thematic synthesis [38]. This qualitative method was a good fit for our research question, which involved a post hoc application of quality-of-care constructs to data collected with the purpose of understanding sIMB-CIM constructs shaping ART adherence in the Haitian context. A codebook was created deductively based on the National Academy’s six dimensions of quality of care as applied to constructs from the sIMB-CIM model. To ensure rigor and reliability, the codebook was independently applied by three analysts to a set of four transcripts in order to identify emergent codes as well as points of divergence indicating need for clarification of the codebook [28]. Upon finalization of the codebook reflecting both deductive parent codes and inductive sub-codes, each transcript was coded independently by one analyst [WD, a Haitian man] and then reviewed by a second coder [NP, a White American female researcher fluent in French with prior experience conducting research in Haiti] to ensure code application consistency and to identify missed codes as well as any further emergent codes. Coders created analytic memos to synthesize themes reflecting dimensions of quality of care. The analysis considered quality dimensions in the context of interactions between patients and HWs, while also considering how these interactions were embedded and shaped within the health system and within the context of community norms. Dedoose software was used in the analysis process [7]. The second coder and lead author [NP] translated into English all quotes selected for inclusion in the paper.

### Ethical approvals

The study received scientific and ethical review and clearance from the University of Washington Human Subjects Division (#52,440) and the Haiti Ministry of Health National Bioethics Committee (#1617–16). All participants in the research provided written informed consent.

## Results

### Participant characteristics

A total of 58 patients, 26 male patients and 32 female, participated in the FGD (Table 2). Among patients, 27 (47%) had either no schooling or primary schooling only, while 31 (53%) had secondary schooling or higher. A total of 57 HWs participated, including 15 nurses, 12 CHWs, 8 health records managers or registration clerks, 5 doctors, 5 pharmacy technicians, 5 laboratory technicians, 4 social workers, and 3 psychologists. Among HWs, 32 (56%) were women, 47 (82%) were aged 35 and above, and 41 (72%) had 5 or more years of professional experience.

**Table 2:** Characteristics of focus group participants

	Port-au-Prince	Cap Haïtien	Total
Patients (*n* = 58 participants)			
Sex			
Female	18	14	32
Male	15	11	26
Age group			
18–25 years	10	2	12
26–35 years	13	6	19
36–55 years	9	15	24
> 55 years	1	2	3
Education level			
No formal education	3	9	12
Primary	6	9	15
Secondary	20	5	25
Superior	4	2	6
Health Workers (*n* = 57 participants)			
Sex			
Female	16	21	37
Male	11	9	20
Age group			
18–25 years	1	0	1
26–35 years	4	5	9
36–55 years	20	24	44
> 55 years	2	1	3
Cadre			
Community health worker	7	5	12
Data clerk	5	3	8
Nurse	5	10	15
Doctor	2	3	5
Pharmacy technologist	2	3	5
Laboratory technician	1	4	5
Psychologist	3	0	3
Social Worker	2	2	4
Duration of service			
< 1 year	0	1	1
1–2 years	5	2	7
3–5 years	1	2	3
> 5 years	21	25	46

### Dimensions of quality in Haitian HIV program

Of the six dimensions of quality, patients and HWs discussed concerns related to safety, patient-centeredness, accessibility and equity (Table 3). Patients described mixed experiences with respect to these quality dimensions, with some reporting highly supportive care and excellent relationships with specific health care providers, and others describing strong concerns that threatened their engagement. Health care workers expressed a strong commitment to supporting their patients and described ways in which they sought to promote patient engagement in care; however, they also conveyed the constraints of providing ART adherence counseling and supportive services in the context of Haiti’s fragile and resource-limited health care system. Table 3 summarizes the themes most salient to each group, with some areas of commonality and several areas of difference. The themes below focus on gaps in quality, and provide a picture of the specific nature of these gaps as experienced by both patients and HW in the embedded context of healthcare in Haiti.

**Table 3:** Key themes related to quality dimensions, from patient and health worker perspectives

**Dimension**	**Patient Perspective**	**Patient**	**Health Worker**
Safety	Risks to privacy in seeking clinic-based HIV supportive services	X	
	Risks to privacy in community-based HIV services	X	
	Lack of care in communication to protect patient privacy		X
	Limited counseling and support on managing ART side effects	X	
	Threats to personal safety of health workers when providing care in the community		X
Patient-centeredness	Challenges with putting the principle of “acceuil” (respectful welcome) into practice		X
	Counseling grounded in standard advice-giving messages rather than personalized needs	X	X
	Uneven trust between patients and providers	X	X
Accessibility, timeliness, affordability	Long clinic waiting times	X	
	Limited help available for patients with financial need	X	
Equity	Material support offered to some patients but not others	X	
	Distrust in fairness of the health system	X	
	Needing an advocate to successfully navigate care	X	X
	Strained working conditions for health workers		X

#### Safety

Safety is defined as “avoiding harm to patients from the care that is intended to help them.” Patients described risks to privacy and confidentiality when seeking care, as well as concerns about side effects of medication and the lack of support in understanding and managing these side effects. In contrast, HW described concerns with their own physical safety while delivering HIV services.

##### Risks to privacy in seeking clinic-based HIV supportive services

Patients spoke repeatedly and in depth and detail about confidentiality concerns. While no patients described blatant privacy breaches, they noted numerous practices that they felt could compromise their privacy, which were problematic in the context of persistent HIV social stigma. For example, one patient described feeling shame in receiving a school fee payment for their child as a social benefit of participating in the HIV program *“This fee payment, they write HIV on it… It is humiliating…. I’m not talking about what they give to the family, it’s what they send to the school office…..as soon as I saw [this]…I ripped the envelope in two and gave it back to them… I’d rather that everyone loses out.”* (*Female patient FGD).* Another patient narrated an experience of attending a support group for ART patients at the hospital, where each person expected to receive a cash incentive for attending the support group. The payment was given as a large bill to one participant, and the group of patients had to seek change from a nearby merchant. Their jockeying about how to divide the payment drew attention of passers-by in the street, which the patient described as a humiliating threat to privacy. Another patient described hesitancy to request a doctor’s note to justify missing work, based on a belief that the note would include documentation of his HIV status.

##### Risks to privacy in community-based HIV services

CHWs noted the importance of carefully managing communication with patients in order to protect privacy: “*We make them our friends and then seek to find a coded way to communicate with code words so that even when other people are around, they will not understand what we are communicating about. We seek to learn about their activities, their religious practices, and all this helps us find them more easily in case of need.” (CHW FGD)* Nevertheless, several patients indicated that they refused to enroll in community-based ART services, because of the risk that CHWs would intentionally or unintentionally reveal their HIV status to family, friends and neighbors. One patient explained: *“As for me, I don’t like when CHWs come to my place, especially when they come often. Neighbors can get the idea that I’m sick….Once I had recently come from the hospital and a CHW came to my place to say the doctor needed to see me even though I had just been there. The worst was that she approached my 11-year old son to ask after me, which made me so mad”(Female patient FGD).* FGDs from both patient and provider perspectives pointed to the importance of balancing efforts to bring patient education, psychosocial support, and care services into the community with efforts to ensure patient privacy.

##### Limited counseling and support on managing ART side effects

HWs described educating patients about side effects as part of the standard practice when conducting “therapeutic education” at the time of ART initiation. They advised patients to expect side effects and to persevere in taking their medicines until their bodies could “get used to” the medicines. Patients experienced a range of ART side effects, including nausea, vomiting, dizziness, headaches, hallucinations, and nightmares. Several described becoming very distressed and frightened by the side effects they experienced, even strongly considering stopping ART due to side effects. While several indicated that HWs helped them understand that the side effects were normal and would diminish with time, noting that this helped them to persist and not abandon using ART, others described turning to advice from peers in order to understand how to cope with side effects. For example, one patient described a fellow patient’s advice to take the ART medications together with sugar water before bed in order to mitigate dizziness and insomnia.

##### Threats to personal safety of health workers when providing care in the community

CHWs described concerns with personal safety when implementing integrated community and clinic-based services. CHWs described navigating out of menacing situations during their community-based work. In an illustrative example, one CHW explained:

*“I needed to contact a sick patient (to bring them their medicines) in a zone that I was not very familiar with…Arriving there, I found people… who seemed to want to harm me…. I was afraid and tried to ask to see the patient’s sister who had accompanied her to the hospital, but she was not home. I decided to leave but the people [in the area of the patient’s home] tried to prevent me from leaving… I was panicking but tried to continue speaking [calmly] with them so they would let me leave… As soon as I climbed on the motorcycle taxi, [the patient’s] father appeared with a machete to harm me. If it wasn’t for the vigilance of the motorcycle taxi driver, who sped away quickly, it could have ended very badly.” (CHW FGD)*

Another female CHW described being harassed by the wife of a patient, who suspected she was the patient’s mistress, to the point that the wife physically followed her and contacted a court to pursue damages against the CHW. Several other CHWs described other stories of being accused and harassed when doing their job in the community, whether delivering medications to patients who enrolled in the community-based ART program, tracing those who were lost to follow up, or checking in on patients between clinical visits. The stories of these challenges underscore the importance of broadening the concept of safety in healthcare to reflect provider safety while delivering services.

#### Patient-centeredness

Patient-centeredness is defined as “providing care that is respectful of and responsive to individual preferences, needs, and values.” Patients and HW both recognized inconsistency in offering a welcoming environment for patients, lack of personalization of counseling, and uneven levels of trust between providers and HW.

##### Challenges with putting the principle of “acceuil” (respectful welcome) into practice

Both patients and providers strongly emphasized the importance of “acceuil”, or having a welcoming, patient-centered atmosphere in the clinic, noting that its absence could de-motivate patients or even drive them away. As explained by a nurse *“We insist on a welcoming atmosphere. It’s important. Having an unwelcome atmosphere can spoil everything. If from the beginning one starts by introducing oneself, getting to know the patient, explaining the services offered, then they will understand.” (Nurse FGD).* However, several patients described being treated brusquely, or with anger or blame. One adolescent female patient expressed her feelings of frustration about how HWs communicate with patients:

“*We need people [HWs] who know how to approach patients, who can receive patients with humor [and] with respect… When [the nurses] give you your paperwork, you can see that they are busy playing with their phones. Yesterday, I was so frustrated I almost cried, but I had no way to speak with them about it because they are older than me… I’m obliged to respect them because they could be my mother or my aunt.” (Female Patient FGD)*

HWs described that time constraints and limited staffing made it difficult to always ensure an atmosphere of “acceuil,” and they acknowledged that some HWs prioritized this more than others.

##### Counseling grounded in standard advice-giving messages rather than personalized needs

Both HWs and patients framed ART “therapeutic education” as grounded in a standardized set of basic messages, including the importance of taking ART at the same time each day whether food is available or not to avoid opportunistic infections, avoiding high-fat and sugary foods, always using condoms when having sex, and avoiding staying up all night or consuming drugs and alcohol. The theme of patients desiring more personalized education about HIV and ART was part of several focus groups, with patients describing wanting more information on how their medications work, about long-term effectiveness and effects of the medicines, and about managing side effects. One female patient described not knowing when and how her newborn infant should be tested for HIV, then being scolded by a health care worker for not bringing her infant for HIV testing at the appropriate time, which was very distressing. Another patient described wanting information about methods for safely conceiving a child, but being hesitant to discuss this with any health care providers. HWs also described typically encouraging ART patients with children to remain adherent in order to survive for many years and be able to support their children’s growth and education, rather than using an approach of exploring salient sources of motivation for each individual. Patients in one focus group noted that the lack of space within the clinic for confidential conversations with providers made it difficult to raise questions and concerns outside of the hearing of fellow patients.

##### Uneven trust between patients and providers

While patients and providers both spoke about specific examples of trustful relationships between individual HWs and patients, there was a sense from patients that these relationships were more the exception than the rule. As expressed by one patient: *“There was an agent [CHW] when I first started in the program whom I really appreciated… when he called me on the phone and reached my husband, he knew how to speak with him and this really pleased me….But now I no longer need an agent! There are lots of unfavorable testimonials about health agents.” (Female Patient FGD)* From their side, HWs described frequent concerns with whether they could trust patient self-reported ART adherence, noting that some patients were suspected to dump or hide pills prior to returning to the clinic for ART refills. As explained by a nurse: *“There was a patient who we followed for a year who said he was 100% adherent but in fact he never took his medicines… Bringing us an empty bottle or almost-empty bottle doesn’t mean that the patient takes the medicine.” (Nurse FGD)***.** In the voice of one patient *“It’s often said that there are three places where as soon as you show up, you are believed to be ‘wrong’: hospitals, prisons, and with ‘hougans’ [traditional healers].” (Male Patient FGD).* The frequently-cited lack of trust between patients and providers directly inhibited patient-centered services.

#### Accessibility

Accessibility refers to minimizing financial, time, and other barriers to care-seeking. Salient challenges to accessibility in the Haitian context included long wait times and lack of financial support for patients in need.

##### Long clinic waiting times

Patient FGDs frequently and passionately raised concerns with clinic wait times. *“When you come here, you can spend the whole day…so the issue of the queue for services needs to be seriously considered, especially when you come to the hospital early having not eaten anything and when you don’t have anything [any money] in your pockets.” (Male Patient FGD)* Another patient noted *“They can have you spend the whole day even though you have other things to do…and the worst is that they are not doing anything more serious than joking amongst themselves, sending telephone messages…” (Female Patient FGD).* Haiti’s MSPP has encouraged service-delivery innovations, such as “fast track” ART refills, and a standard that HIV clinic visits should not exceed 1.5 h; however, providers noted challenges when personnel get pulled away from the clinic for meetings, trainings, or errands and challenges with shifting patient expectations away from a pattern of arriving early in the day and queue for visits rather than coming at specific appointment times.

##### Limited help available for patients with financial need

Financial barriers to engagement in care were a frequent theme in both patient and provider focus groups. They noted patient responsibility to pay for tests, radiography, and some medicines not offered for free through the ART program. They also discussed how reimbursement for transportation was only sometimes offered, and how the amounts offered were insufficient to cover costs for those coming from outlying areas. Some patients looked to providers for individual favor and help. One patient expressed a sense of disappointment and frustration: *“Even when you tell the nurses you have nothing to eat with your medicines, they’ll tell you to take the medicines in spite of everything, they’ll never give you even 50 gourdes [about $0.40 USD] to help you.” (Female Patient FGD)* However, another patient affirmed that HWs should not be expected to offer material support: *“In my opinion, those who we believe should be capable of helping us economically often have a lot of responsibility, so it’s up to us to take care of ourselves and money is not the most important thing. I remember having a fungal infection that almost left me blind…but I knew that I had to take care of myself and use the medicines the doctors gave me.” (Female Patient FGD)* In the context of Haiti’s culture of “patronage” where the poor and powerless cultivate connections with powerful people who can look out for them, HWs described the strain and stress of facing patients who sometimes expected or hoped that HWs would offer financial assistance “from their own pockets.”

#### Equity

Equity refers to care that does not vary in quality because of patients’ personal characteristics. Patients expressed concerns that HW attention and healthcare resources were differentially directed to patients with higher status or connections, and both patients and HW noted the importance of having an advocate within the system to successfully navigate care. HW noted highly challenging working conditions, including delayed salary payments.

##### Material support offered to some patients but not others

Patients expressed strong concerns about equity of distribution of material support associated with the HIV program. For example, one patient noted: “*There is an issue with discrimination here…for example, if they see you as having a normal, healthy appearance, they will not give you food even though they know that if you are taking medicines you will appear normal and healthy.” (Female Patient FGD)* Another patient suggested that high-value resources are unfairly withheld from certain patients:

*“Poor people can’t get [food supplements] anymore. Foods with protein to eat along with the medicine are there, but they don’t want to give them out. They don’t give you any liquid medicines or anything with iron. If the medicine is sold for more than 50 Haitian dollars, they won’t give it out…One day, there was no Vitamin C syrup for me, but when a ‘big person’ comes, they give it to them. When it’s their friends who come, they give it out.” (Female Patient FGD).*

Patients in multiple FGDs spoke about how different health facilities offered different social benefits associated with HIV services (e.g. food supplements, transportation vouchers, incentives for participating in support groups). That the availability of these benefits was variable over time and unpredictable seemed to breed distrust in the broader system of HIV care, based on lack of transparency. Building further transparency about eligibility criteria for material support or advocating for universal access to a minimum package of material support could help to build patient trust in the fairness of the system.

##### Distrust in fairness of the health system

In addition to concerns with equity of services at an individual, interpersonal level, patients also expressed concerns with the equity and justice of the system at a broader, more abstract level. In several FGDs, patients suggest power and profit motives in the health system. For example: *“I have heard it said that there is a lucrative market around this issue of AIDS and that the researchers are not interested in finding a definitive treatment. This issue of AIDS has been around for too long. I’ve told the doctors to not hide the cure from us if they have one.” (Female Patient FGD)* This quote demonstrates the abstract sense that the system is not set up to serve the powerless average person, but is manipulated by the powerful for their gain, and that the only recourse at the individual level is to appeal to an individualized, favored patronage relationship for help.

##### Needing an advocate to successfully navigate care

Several patients described the need to form a personal alliance with a HW, in order to get that individual’s help them navigate the clinic circuit and get services in a timely way. One HW described going to extra lengths to help patients who they believed were at risk of defaulting: *“Some people are very sensitive, as soon as they see people lining up in front of them, they may give up and go home…We can sometimes go to the reception desk and tell them to look out for this or that patient, out of fear that they leave and don’t return.” (Doctors FGD).* However, this strategy of individualized advocacy to help navigate the system could be seen poorly by other patients who were not similarly favored. As one patient noted: *“Once I came here really early, and there were three men accompanied by a beautiful woman. I was here early, but they let me wait. One was a police officer, and they gave him his medicines. But those who come, who are not police officers, get nothing. You may come at 7 am and still wait until 2 pm without being called.” (Female Patient FGD)* Both patients and HWs described cultivating and enabling favored relationships to help specific individuals navigate a system marked by scarcity, but overall this came at the cost of perceived fairness.

##### Strained working conditions for health workers

HWs noted strong concerns with the fairness of the health system in offering them a supportive work environment. They described that in the recent past they had endured periods of 4–5 months with delayed salary payments, yet faced the expectation that they come to work even when not being paid at regular intervals. At the time of the study, there was a strike of janitorial and support staff in the public health system that resulted in the closure of many primary care services, and crossing the strike line through groups of demonstrators came with some risk of physical injury. These strained working conditions made it challenging for HWs to focus on providing patient-centered care.

## Discussion

The FGDs with patients and HWs involved in Haiti’s national ART program highlighted concerns with several quality dimensions, including safety, person-centeredness, accessibility, and equity. While patients cited examples of positive patient – provider interactions for patient education, adherence counseling, and psychosocial support, they also noted multiple modifiable concerns that inhibited trust and satisfaction with services. HWs noted how difficult working conditions challenges their ability to provide patient-centered services. In particular, we found that both HWs and patients used strategies of forging favored relationships to help navigate a challenging health care system. However, these favored, patronage-based relationships came at the cost of perceived fairness for average patients without these special relationships, as well as pressure on HWs to help individual patients from their “own pockets.” These concerns reflect the embeddedness of patient – provider interactions within a health care system marked by scarcity, power dynamics between patients and providers, and social stigma related to HIV. Hanefeld et al. [18] describe this type of “embeddedness”—how individual patient experiences in interacting with HWs over time take place within the context of broader health system structures and community norms—as an important challenge in conceptualizing and measuring quality of care [18].

Our study contributes to a broader literature on patient satisfaction in HIV care and treatment programs in LMICs. Our findings on the salience of respectful, kind providers and privacy in ensuring patient satisfaction were consistent with research from a large patient satisfaction survey in Nigeria [4], and from several discrete choice experiments in Zambia and Zimbabwe showing that preferences for “respectful and understanding” or “nice” personnel dominated in importance over other features of care like availability of community-based ART, proximity to the clinic and even clinic waiting times [42, 48]. In Tanzania, patients rated quality of care and respectful staff as key reasons to travel to more distant health care sites [15]. Our findings on equity, trust, and accessibility issues also echo themes found in prior qualitative research in other LMICs. For example, a study in South Africa found similar concerns with preferential treatment of friends and relatives, unprofessional conduct (especially for CHWs), long wait times, and transport costs [3]. In Malawi, patients reported distrust of HWs based on suspicions they misused ART drug stocks for personal gain [34].

Our study makes several unique contributions to the literature on quality of care. First, it demonstrates particular concerns with safety, patient-centeredness, accessibility, and equity in the Haitian context, bringing these concerns to life and complementing prior assessments focused on structure (e.g. the availability of infrastructure, equipment and supplies for delivering services) and process (e.g. HW knowledge, compliance with guidelines) through Service Provision Assessment (SPA) surveys in Haiti. For example, Haitian HWs faced consistent pressure to offer support to patients “from their own pockets” and clients sought privileged advocacy by health workers within the system, reflecting a broader social reality of poverty, lack of a social safety net, and income inequality and a “culture of domination” where social mobility often depends on personal connections and networks of patronage [8, 16]. For HWs, this expectation of patronage led to job stress, even as the lack of a routine, standardized package of support for patients with severe financial need limited the accessibility of services.

Our study contributes to prior qualitative research in LMICs exploring HW perceptions of quality of care and their own roles in contributing to patient satisfaction. In South Africa HWs noted the lack of health system resources (e.g. staff shortages, medication stock-outs, and limited or unsuitable clinic space) as well as their own lack of training, supervision and positive reinforcement (including bonus payments) as inhibitors of HIV care quality [24]. In Zambia, HWs spoke about emotional and cognitive barriers to providing patient-centered care such as beliefs that “punishment works” to motivate patient compliance, that provider authority should be reinforced rather than emphasizing empathy or shared decision-making, that patients cannot be trusted, and that they are overwhelmed and tired and unable to do more to cater to patients [5]. While some of these themes echo those in our research, we also found the striking importance of threats to personal safety (especially for CHWs) and chronic delays in salary payments. In Haiti, low wages, delays in salary payments, and poor working conditions have led to HW strikes in the public sector, resulting in reduced availability of basic health services [19]. Our findings fit within a broader literature demonstrating the role of low and/or delayed wages in contributing to HW absenteeism, dual practice (holding more than one job), low motivation, predatory behavior, and turnover [1, 13, 43].

A unique contribution of our research is that it considered both patient and HW perspectives. Patients and HWs each discussed themes related to quality constructs with some areas of overlap but many points of difference. Both noted the use of standard advice rather than personalized counseling, lack of trust between patients and HWs, limited help for patients with financial need, and the importance of patients having a personal advocate from within the system to navigate care. Patients had salient concerns around risks to privacy, lack of support on managing ART side effects, long clinic wait times, and lack of equity and fairness in the health system. In contrast, HWs perceived that they generally took care to protect patient privacy. HWs noted the strained working conditions that made it difficult for them to offer respectful, welcoming services. This contrast in perspectives underscores that addressing working conditions for HWs will be necessary in efforts to improve patient experience of quality; however, topics like HW safety and inconsistent salary payments receive limited treatment in the National Academies quality framework [32].

Key recommendations for the health system in Haiti arising from our analysis include: the need to balance efforts to improve ART accessibility through community-based ART services with efforts to protect patient privacy; the need to increase patient-centeredness in education and counseling, going beyond standard advice-giving messages to address the particular questions and concerns of each individual; the need to improve transparency around criteria for access to incentives and material support to build patient trust in fairness and equity of services; and the need to attend to improve working conditions for HWs, including timely payment, physical safety, and mitigating expectations that HWs should use their personal financial resources to support individual patients.

### Strengths and limitations

A strength of our study was that it assessed the perspectives of both patients and HWs using an open-ended approach to inquiry about patient education, counseling and psychosocial support services within Haiti’s ART program, which permitted nuanced insights about quality dimensions. The engagement of Haitian FGD facilitators allowed participants to express themselves openly, and the triangulation of perspectives of two analysts coding the transcripts was also a study strength. Limitations of the study included the fact that the FGDs addressed a range of themes related to the formative design of the *InfoPlus Adherence* intervention, meaning that there was limited time to explore quality of care. Background traffic noise interfered with interpretation of certain parts of the FGD recordings. Participants should not be seen as representative of all patients and HWs in the country.

## Conclusion

The past two decades have brought extensive investments to strengthen health systems in LMICs, with particular focus on investments in HIV programs. While Haiti has participated in SPA surveys addressing structure and process indicators of quality, there is less evidence about patient and provider perceptions of quality in the context of longitudinal patient-provider interactions. This qualitative study highlighted modifiable aspects of safety, patient-centeredness, accessibility and equity of patient education, counseling and psychosocial support services within Haiti’s ART program. Reinforcing key aspects of HIV care quality –including a respectful and welcoming atmosphere, timely service, privacy protection, and building patient perception of fairness in access to support—could help to reinforce patient satisfaction and care engagement in Haiti. At the same time, improving working conditions for HWs will be a critical step in order to achieve quality.

## Data Availability

The datasets used and/or analysed during the current study are available from the corresponding author on reasonable request.
